# High mortality after ST-segment elevation myocardial infarction and implications for implantable cardioverter defibrillator trials design

**DOI:** 10.1093/europace/euaf096

**Published:** 2025-05-06

**Authors:** Laurent Fauchier, Alexandre Bodin, Bertrand Pierre

**Affiliations:** Service de Cardiologie, Centre Hospitalier Universitaire Trousseau, Avenue de la Republique, Tours 37044, France; Faculté de Médecine, Université François Rabelais, 2 boulevard Tonnellé, Tours 37000, France; Service de Cardiologie, Centre Hospitalier Universitaire Trousseau, Avenue de la Republique, Tours 37044, France; Faculté de Médecine, Université François Rabelais, 2 boulevard Tonnellé, Tours 37000, France; Service de Cardiologie, Centre Hospitalier Universitaire Trousseau, Avenue de la Republique, Tours 37044, France; Faculté de Médecine, Université François Rabelais, 2 boulevard Tonnellé, Tours 37000, France

We read with interest the article by Stødkilde-Jørgensen *et al*.^1^ on mortality and use of implantable cardioverter defibrillators (ICDs) in patients following non-ST-segment elevation myocardial infarction (NSTEMI). This nationwide Danish cohort study highlights the persistently high mortality among NSTEMI patients with left ventricular ejection fraction (LVEF) ≤ 35% at the index procedure. Remarkably, ICD implantation rates remained low in this high-risk group despite possible guideline eligibility.

We recognize the specificities of the Danish dataset—including the absence of data on LVEF recovery at 3 months, optimal medical therapy (OMT), New York Heart Association class, and cause of death. The absence of cause-of-death data—particularly regarding sudden cardiac death—limits interpretation of whether ICD therapy might have altered outcomes and underscores the need for caution when using these findings to inform preventive strategies. The findings nonetheless raise important questions for the design and interpretation of ongoing ICD trials, notably the PROFID-Reduced study.^2–4^ The PROFID excludes patients within the first 3 months post-MI and requires symptomatic heart failure, stable LVEF ≤ 35%, and confirmed OMT. Thus, the Danish study and PROFID address related but distinct populations. The underlying assumption of PROFID is that advancements in pharmacologic management have significantly reduced sudden cardiac death risk, potentially rendering ICD therapy unnecessary in many patients.^5–7^

Nonetheless, the persistently elevated mortality seen in the Danish cohort—44% at 5 years—highlights the continued vulnerability of patients with ischaemic cardiomyopathy. Although the study lacks cause-of-death adjudication, the persistently high mortality remains a major concern. Historical data suggest a significant proportion of these deaths may be arrhythmic and potentially preventable by ICD therapy.^5,8^ This serves as a cautionary signal when interpreting non-inferiority frameworks that test the omission of ICD therapy in similar subgroups. Although a non-inferiority design is methodologically appropriate for evaluating modern ICD utility, the choice and communication of the non-inferiority margin are critical. A 2021 publication regarding the non-inferiority PROFID-Reduced trial references a margin corresponding to an upper hazard ratio boundary of 1.336,^3^ but detailed justification remains limited in public sources. Transparency in statistical assumptions is essential to ensure clinical and ethical acceptability, especially when outcomes may include preventable arrhythmic death.

We also advocate for prespecified subgroup analyses within PROFID, particularly regarding NSTEMI vs. STEMI presentations. These groups may differ in arrhythmic substrate and competing risks. Such stratification could refine our understanding of ICD efficacy in specific clinical contexts.

Finally, the limitations of LVEF as the sole stratification tool are increasingly evident.^9,10^ While LVEF remains central to patient selection per guidelines, the PROFID platform may present an opportunity to explore and potentially validate additional risk stratifiers, including imaging, biomarkers, and ECG-based tools. These tools could inform future strategies for selecting patients most likely to benefit from ICD therapy.

In summary, although the Danish cohort differs in structure from PROFID, it underscores ongoing mortality in high-risk patients and illustrates the importance of methodological rigour, subgroup evaluation, and transparency in ICD trial design. These considerations are essential to support ethically sound and clinically relevant hypotheses and conclusions for future trials.

## Data Availability

No new data were generated or analysed in support of this research.
